# Ribo-attenuators: novel elements for reliable and modular riboswitch engineering

**DOI:** 10.1038/s41598-017-04093-x

**Published:** 2017-07-04

**Authors:** Thomas Folliard, Barbara Mertins, Harrison Steel, Thomas P. Prescott, Thomas Newport, Christopher W. Jones, George Wadhams, Travis Bayer, Judith P. Armitage, Antonis Papachristodoulou, Lynn J. Rothschild

**Affiliations:** 1 Department of Biochemistry, University of Oxford, South Parks Road, Oxford, OX1 3PJ UK; 2 Department of Engineering Science, University of Oxford, Parks Road, Oxford, OX1 3PJ UK; 3 Asimolar Bio, Inc., 953 Indiana St, San Francisco, CA 94107 USA; 4 NASA Ames Research Center, Mail Stop 239-20, Moffett Field, CA 94035 USA

## Abstract

Riboswitches are structural genetic regulatory elements that directly couple the sensing of small molecules to gene expression. They have considerable potential for applications throughout synthetic biology and bio-manufacturing as they are able to sense a wide range of small molecules and regulate gene expression in response. Despite over a decade of research they have yet to reach this considerable potential as they cannot yet be treated as modular components. This is due to several limitations including sensitivity to changes in genetic context, low tunability, and variability in performance. To overcome the associated difficulties with riboswitches, we have designed and introduced a novel genetic element called a ribo-attenuator in Bacteria. This genetic element allows for predictable tuning, insulation from contextual changes, and a reduction in expression variation. Ribo-attenuators allow riboswitches to be treated as truly modular and tunable components, thus increasing their reliability for a wide range of applications.

## Introduction

**Figure 1:**
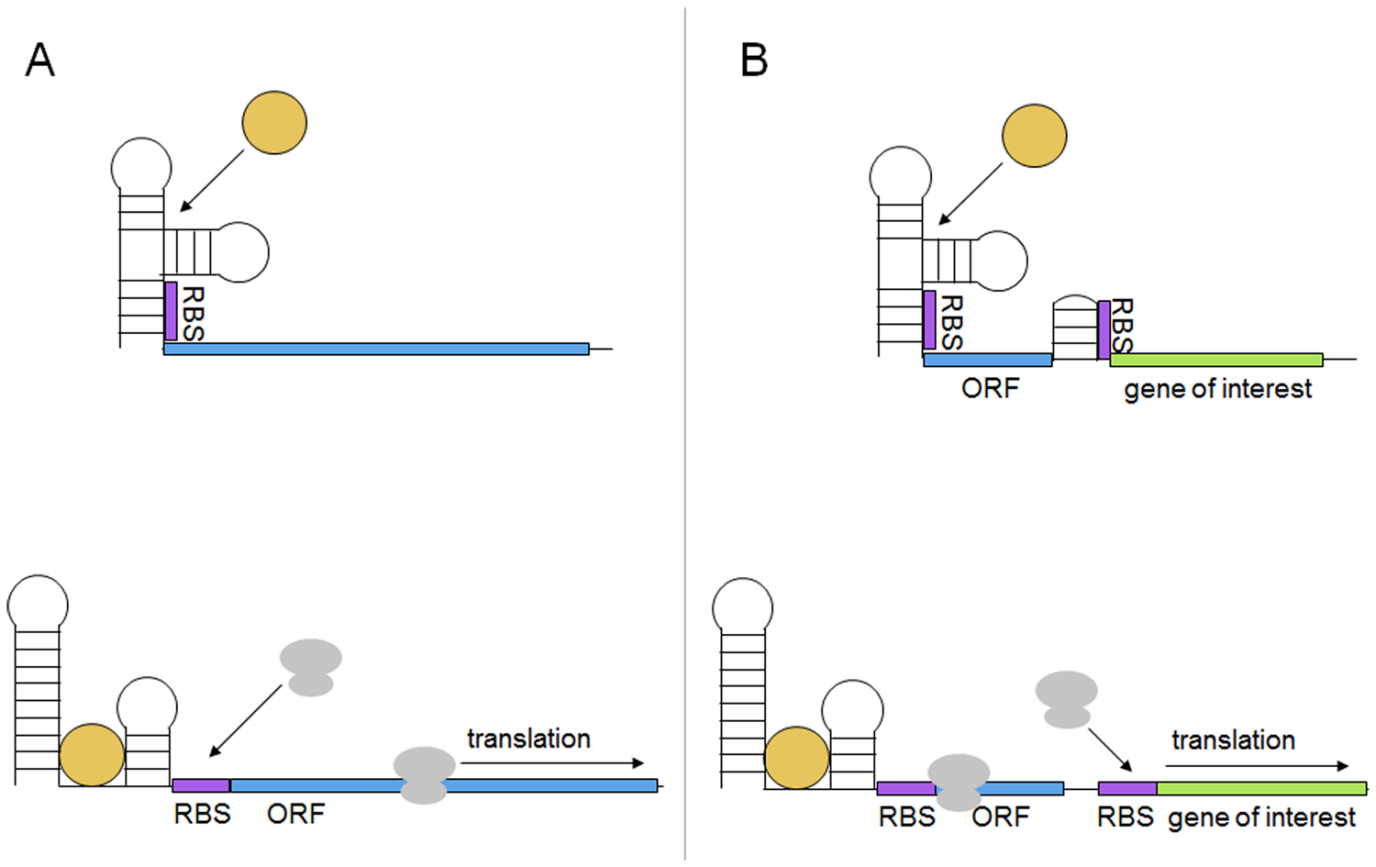
Typical activating riboswitch function. A ribosome binding site (RBS, purple) is sequestered within a riboswitch preventing ribosome recruitment. (**A**) A standard riboswitch in which binding of a ligand (yellow) causes a conformational change exposing the RBS, allowing translation of the gene of interest (blue). (**B**) A ribo-attenuator adds a second RBS, sequestered away by a local hairpin. The hairpin can be opened by a ribosome travelling from the riboswitch RBS, exposing the attenuator RBS and allowing translation of the gene of interest. Dynamic operation of both systems is further explained in our video animation^53^.

Riboswitches are structural regulatory elements generally found in the 5′ UTR of messenger RNA which allow the regulation of a downstream gene or operon in response to the binding of small molecules such as cellular metabolites or metal ions^1–5^. Riboswitches can regulate a variety of processes including transcription, translation, and splicing in eukaryotes^6^. Riboswitches that regulate translation do so through the allosteric effects of small molecules binding to their aptamer domain. This causes a structural rearrangement which usually opens up or sequesters away a ribosome binding site (RBS) core (Fig. 1A)^7–9^. As riboswitches rely exclusively on RNA for structure and are present in all three domains of life, it is possible that they arose as an early regulatory element in the hypothesized RNA-based world^10^.A range of riboswitches evolved to control the expression of enzymes in natural systems, from which synthetic riboswitches have been adapted to respond to specific molecules^11–16^.

To be truly useful for synthetic biology, riboswitches should be modular “plug and play” devices. Despite significant promise and over a decade of research, this is still not the case. For example, many riboswitches selectively bind a small molecule of interest using an aptamer whose specific RNA secondary structure is influenced not only by its own sequence, but also by the surrounding genetic context including the proximal open reading frame (ORF) under the control of the riboswitch. Thus, substituting the original ORF with a new one on a ‘start codon for start codon’ basis can nullify the desired riboswitch response to a given ligand^17^. To overcome this lack of modularity, many studies have created fusions comprised of a riboswitch, the first few hundred base pairs of its working ORF, and a gene of interest^18–21^. However, this approach fails in many circumstances as it can alter the gene’s functionality, since many enzymes, such as the endoribonuclease XendoU, will not work with the inclusion of large 5′ fusions^22–27^.

**Figure 2:**
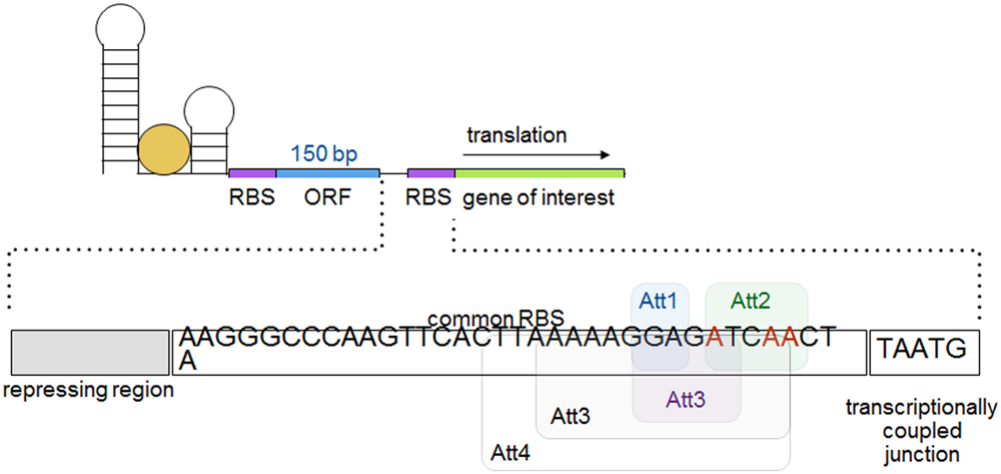
Ribo-attenuator context and schematic: A ribo-attenuator (Att) is inserted after a riboswitch and 150 bp of its native ORF, and precedes the desired gene of interest (GOI). We defined a ribo-attenuator element to include a repressing region, a common RBS region (within which the RBS is underlined), and a transcriptionally-coupled junction. The repressing region, which for all attenuators except Att2 is the reverse complement of a subsequence (enclosed by the labelled boxes) of the common RBS region (see Supplementary Table 2 for sequences), is selected so that the attenuator region as a whole forms a hairpin with appropriate ∆G (see Supplementary Fig. 1 for predicted conformations). For Att2 no repressing region was included. Instead, bases highlighted in red were altered to encourage formation of a hairpin (which involved the sequence indicated) downstream of the RBS.

Despite advances towards the *in silico* design of riboswitches^28–30^, adjusting their functional range in response to a given ligand remains challenging. This difficulty arises because a riboswitch’s activation or repression response is determined by both its RBS strength and the secondary structure surrounding its aptamer domain (which is itself dependent on the RBS sequence)^30^. Thus, due to the poor understanding of *in vivo* RNA structures changing a riboswitch’s induction range via alteration of the RBS (generally a more predictable approach than promoter modification) without destroying the induction response is difficult. Further limitations to application of many synthetic riboswitches arise due to variation in the expression of genes under their control, which is thought to originate from ligand-dependent RBS accessibility bursts^7^. This variation limits their use with many high throughput screening techniques (such as Fluorescence Activated Cell Sorting (FACS) sorting) that rely on a distinct separation between a positive and negative population. Several solutions to these individual problems have been suggested. For example, the use of T7 polymerase could amplify the output of the riboswitch and improve its functional range^31^, however, its application is limited by its reliance on low basal expression, and does nothing to decrease expression noise. To overcome the limitations of large 5′ fusions, fixed induction ranges, and sensitivity to ORF changes of both engineered and natural riboswitches, we designed and tested ribo-attenuators (Atts). These novel genetic elements are placed after 150 base pairs of a riboswitch’s working ORF. They consist of a hairpin containing a RBS on the downstream portion of the stem in order to silence translation independent of upstream riboswitch activity, followed by a negative one shifted transcriptionally-fused stop and start codon (TAATG) (Fig. 2). The passage of ribosomes recruited by the upstream riboswitch opens up the introduced hairpin, before dissociation at the proximal in-frame stop codon (TAA) in the transcriptionally-fused junction. Additional ribosomes can then assemble at the ribo-attenuator RBS and initiate translation at the first start codon of the introduced gene of interest (Fig. 1B). Therefore, instead of directly controlling the translation of a gene as in its natural setting, the riboswitch instead controls the translational initiation rate from the downstream attenuator RBS. As such ribo-attenuators facilitate tuning of a riboswitch’s response, the orthogonal expression of a novel gene of interest, and by improving the reliability with which the downstream RBS remains exposed, they also reduce expression variability within populations. Ribo-attenuators thus overcome many of the issues preventing widespread use of re-engineered or synthetic riboswitches.

In this paper we employ two riboswitch systems; the activating addA riboswitch from *Vibrio vulnificus* ^15^ that binds 2-aminopurine, and the repressing btuB riboswitch from *Escherichia coli* ^18^ that binds adenosylcobalamin. For both of these riboswitches we demonstrate that replacement of their working ORF results in a loss of ligand response, though this can be overcome by fusing an introduced gene-of-interest (GOI) to 150 bp of their working ORF. We then design and test nine potential ribo-attenuators within the addA riboswitch system, from which we select five attenuators that give a range of output responses. These five attenuators are used with the addA riboswitch to express sfGFP and OmpT, demonstrating that ribo-attenuators can reduce expression variability (which we support with a theoretical model), avoid build-up of inclusion bodies, and (in the case of OmpT) achieve proper targeting of membrane proteins. The same five attenuators are then modularly inserted into the btuB riboswitch system. These are used to express eGFP and ColE9, demonstrating again that ribo-attenuators can reduce expression noise, and that they allow amplification and tuning of a riboswitch’s output response. Our experimental results thus support the theoretical mechanisms that motivated the design of ribo-attenuators, and demonstrate that ribo-attenuators can be modularly employed in different riboswitch systems to control the expression of a range of functional outputs.

## Materials and Methods

### Plasmids

All riboswitch cassettes were cloned into the J64100 plasmid^32^ (ColE1 origin; 50–60 copies, chloramphenicol resistance) under control of the tetracycline promoter. The addA riboswitch sequence was the kind gift of Dr Neil Dixon (Department of Chemistry, University of Manchester) and the btuB riboswitch was amplified from the *Escherichia coli* genome. All riboswitches were synthesised by Integrated DNA Technologies (IDT; Skokie, IL, USA) as gBlock gene fragments, assembled by Gibson assembly^33, 34^ and the sequence confirmed. A full list of plasmids is provided in Supplementary Table 1, full plasmid sequences are available from (37). A list of ribo-attenuator sequences is provided in Supplementary Table 2.

### Growth and Induction

All experiments were performed in *E*. *coli* DH5*α*Z1^35^. For each experiment, three freshly transformed colonies were inoculated in LB medium (10 g L^−1^ tryptone, 5 g L^−1^ yeast extract, 10 g L^−1^ NaCl) with chloramphenicol (25 µg mL^−1^) and grown overnight at 37 °C with shaking. For the functional assay and riboswitch characterisation, overnight cultures were diluted 1:100 into 1 mL LB in a deep well plate (M9810-50EA, Greiner Bio-One, Stonehouse UK) with chloramphenicol (25 µg mL^−1^), anhydrotetracycline (150 ng mL^−1^) and stated concentrations of riboswitch inducer. For protein assays, overnight cultures were diluted 1:50 into 50 ml LB with the same antibiotic and inducer concentrations. Cultures were induced for 8 hours at 37 °C with shaking before data collection or harvesting for protein analysis. Biological triplicates were generated to assess behavioural variations between cultures.

### Data Collection

After induction, 500 µL of each culture was centrifuged at 5300 × g, washed with PBS and resuspended in 500 µL of PBS. OD_600_ measurements were taken in clear 96-well plates and GFP measurements in black well plates (M5061, M2936, Greiner Bio-One, Stonehouse UK) in a CLARIOstar platereader (BMG Labtech, Ortenberg, Germany). GFP expression was quantified by normalising by OD_600_. Flow cytometry was performed on an Attune flow cytometer (Lifetech Scientific, Basingstoke, UK) for each data point and 50,000 events were measured. Raw data for bar graphs are available online^36^.

### Fluorescence Microscopy

Fluorescence microscopy was performed on cells grown and induced as described above. Thin, agarose pads (1% w/v agarose in Milli-Q water) were generated on microscope slides. Two µL of cells were added to the pad, immobilising them on the surface of the agarose. Cells were then imaged with a Nikon Eclipse Ti microscope (with NIS Elements software), 100 × phase contrast objective (Nikon), GFP filter set (Chroma), and images collected with an Andor iXON CCD camera.

### Cell disruption

Cells were harvested (3700 × g, 30 min) and pellets were re-suspended in Lysis Buffer (PBS-Buffer with 137 mM NaCl, 2.7 mM KCl, 10 mM Na_2_HPO_4_, 1.8 mM KH_2_PO_4_, pH 7.4, with addition of 0.2 mg ml^−1^ lysozyme (Sigma Aldrich), 20 µg ml^−1^ DNAse (Sigma Aldrich), and 1 protease inhibitor tablet per 50 ml solution (Complete EDTA free protease inhibitor, Roche)) relative to the wet weight of the cell pellet (1 mL buffer per 50 mg pellet). Cells were disrupted using a fast prep device (2 × 6 m s^−1^, 30 s, FastPrep-24 5 G, MP Biomedical) or sonication (1 min total, 10 s on, 4 s off, 50%, Vibra-Cell VCX130, Sonics). The insoluble material was pelleted by centrifugation at 8,000 × g (OmpT) or 20,000 × g (ColE9), for 20 min.

### Functional assay of ColE9

Supernatants of the lysed cells were separated and diluted from 10^0^ … 10^10^ in PBS buffer. Their potency was analysed by a plate killing assay^37^. An LB plate containing 0.8% agar was warmed to 42 °C before an established culture was added at a dilution of 1:10,000. The plate was poured and left to dry for 30 min at 37 °C then 2 µL of the supernatants at the given dilutions was spotted on a plate and incubated at 37 °C for 16 h. The potency of ColE9 was assessed by observing the clearance around each spotted point on the plate.

### Preparation of strep-tagged OmpT

After cell disruption, the supernatant containing membrane-bound OmpT and the pellet containing improperly folded OmpT were treated as follows. To precipitate the membranes, the supernatant was spun down (120,000 × g, 1.5 h) and membranes were re-suspended in 50 µL PBS. To extract membrane-bound OmpT, the re-suspended membranes were diluted with 50 µL PBS containing n-dodecyl-*β*-D-maltopyranoside (Anatrace) giving a final concentration of 0.635 mM (5× the critical micelle concentration where 1 CMC is the concentration of surfactants above which micelles form). The extraction was performed overnight by shaking at 4 °C, and the insoluble material was pelleted (12,000 × g, 20 min) yielding soluble OmpT. The remaining pellets harbouring OmpT as inclusion bodies (IB) were washed as described previously^38^ using PBS with 1% Triton-X-100 for the removal of residual *E*. *coli* lipids. Washed IB were dissolved using 6 M gunanidinium chloride in PBS and incubated for 10 min at 95 °C. Insoluble material was removed (12,000 × g, 5 min) yielding the IB fraction. Whole cells (WC), extracted membrane bound OmpT (Mem) and solubilized misfolded OmpT (IB) were resolved with 4–20% SDS-PAGE (Mini-PROTEAN TGX, BIORAD), using the Laemmli buffer compositions^39^. Immunoblotting was performed as described previously^40^ using anti-strep antibodies (ThermoFisher Scientific) and Luminata Western HRP Substrate (Merck Millipore).

### Model Methods

The cumulative distribution of the random variable T denoting the time elapsed between an arbitrary time-point *τ* and the next production of a GFP molecule was calculated according to the model described in the Supplementary Information. This model treats the transition between closed (OFF) and open (ON) states of both the riboswitch and ribo-attenuator as stochastic random processes, with the rate of attenuator opening taking an increased value when the riboswitch is ON. This dependency captures the system’s intended mechanism, whereby a ribosome initiated at the riboswitch RBS opens the attenuator, revealing the second RBS. The primary assumptions made are therefore the model’s parameterised rate values, for which we used the following:

1 $$\begin{array}{rcl}\lambda (ON) & = & 50,\,\mu (ON)=4,\lambda (OFF)=\mu (OFF)=0.01\,{K}_{-}=0.5\,{m}_{-}=1\,\\ {K}_{+[I]} & = & \frac{{[I]}^{2}}{{[I]}^{2}+{[10]}^{2}}\end{array}$$

For 0 ≤ [*I*] ≤ 50 (where [*I*] = 50 corresponds to 100% induction. The inverse of T is the random variable we call “Expression Rate”. For the calculation of the CDFs, see Supplementary Information and the corresponding MATLAB code.

## Results

### Change in open reading frame demonstrates sensitivity of 5′ regulatory elements to secondary structure

**Figure 3:**
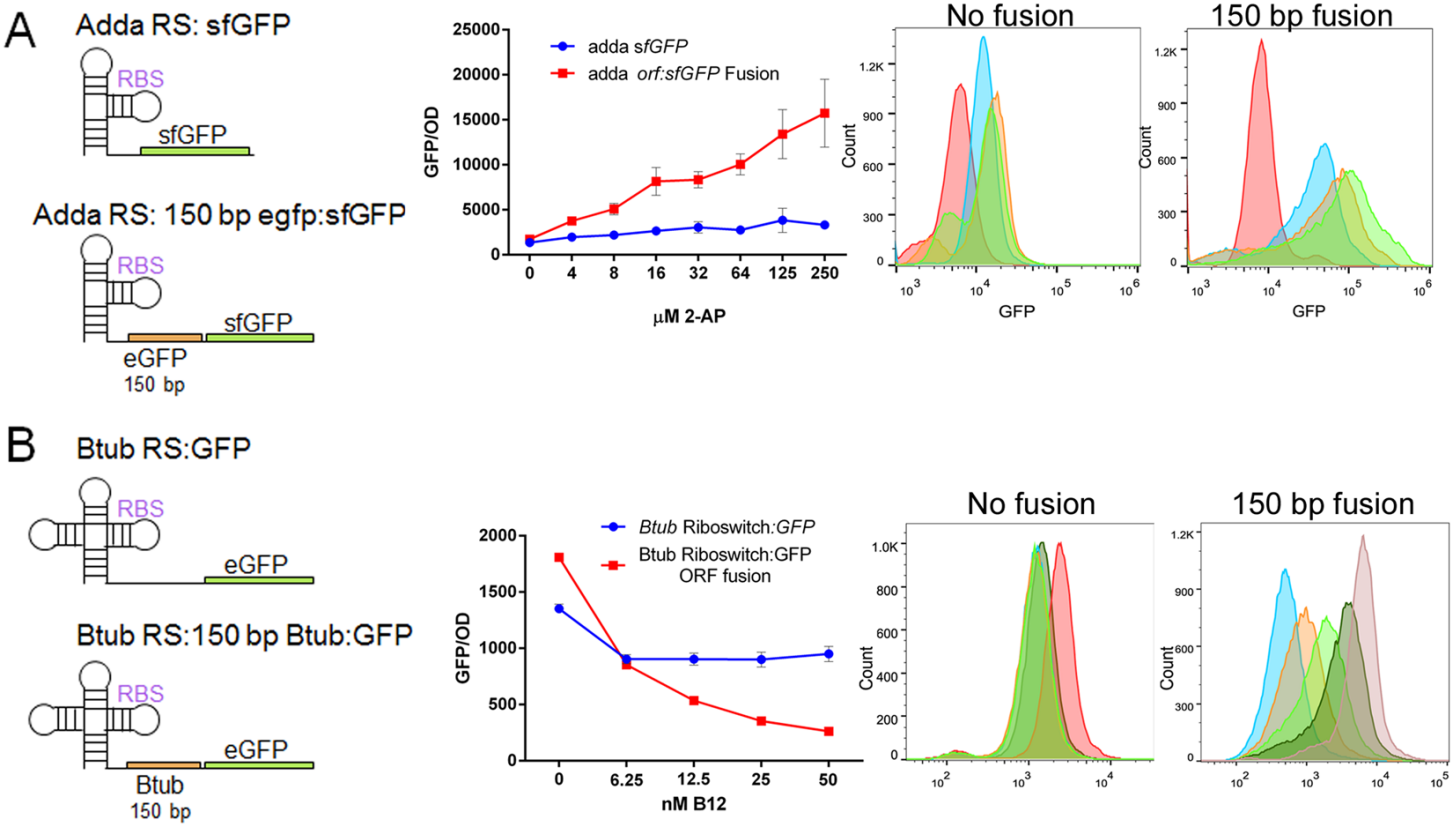
AddA and btuB riboswitches exhibit contextual sensitivity. (**A**) The adenine riboswitch had sfGFP introduced directly after the riboswitch replacing its reported ORF (previously eGFP), and also fused to the first 150 bp of the previous ORF. Both constructs were analysed for population and single cell response to 2-aminopurine. Error bars indicate standard deviation of measurements for biological triplicates. Single cell colours; Red (0 µM) Blue (8 µM) Orange (32 µM) Green (250 µM). (**B**) The btuB riboswitch had eGFP introduced directly after the riboswitch replacing its reported ORF (previously btuB), and also fused to the first 150 bp of the previous ORF. Both constructs were analysed for population and single cell response to adenosylcobalamin. Single cell colours; Red (0 nM) Dark Green (6.25 nM) Light Green (12.5 nM) Orange (25 nM) Blue (50 nm).

Two previously reported riboswitches were investigated for sensitivity to changes in the ORF. The addA riboswitch, characterised from *Vibrio vulnificus* ^15, 41^, is an activating riboswitch reported to selectively bind 2-aminopurine^42–44^. We fused sfGFP^45^ to the first 150 base pairs of the previously reported working ORF^15^, which yielded an induction response (to 2-aminopurine concentrations between 0 and 250 µM) very similar to previously reported studies^15^. However, replacing the entirety of the riboswitch’s reported ORF with sfGFP gave no induction response. These data were supported by single cell analysis, which demonstrated a response to the inducer for the fusion construct, but no such output when the ORF was completely substituted (Fig. 3A).

The second riboswitch investigated is the btuB riboswitch, a repressive riboswitch notable for its response to the small metabolite adenosylcobalamin, an active form of vitamin B_12_^18^. As with the addA riboswitch, replacing the working ORF with eGFP^46^ resulted in a loss of induction response (between 0 and 50 nM of adenosylcobalamin) for both colony and single-cell measurements. However, fusion of eGFP to 150 base pairs of the working ORF yielded the desired repressive induction response (Fig. 3B).

### Design of ribo-attenuators

**Figure 4:**
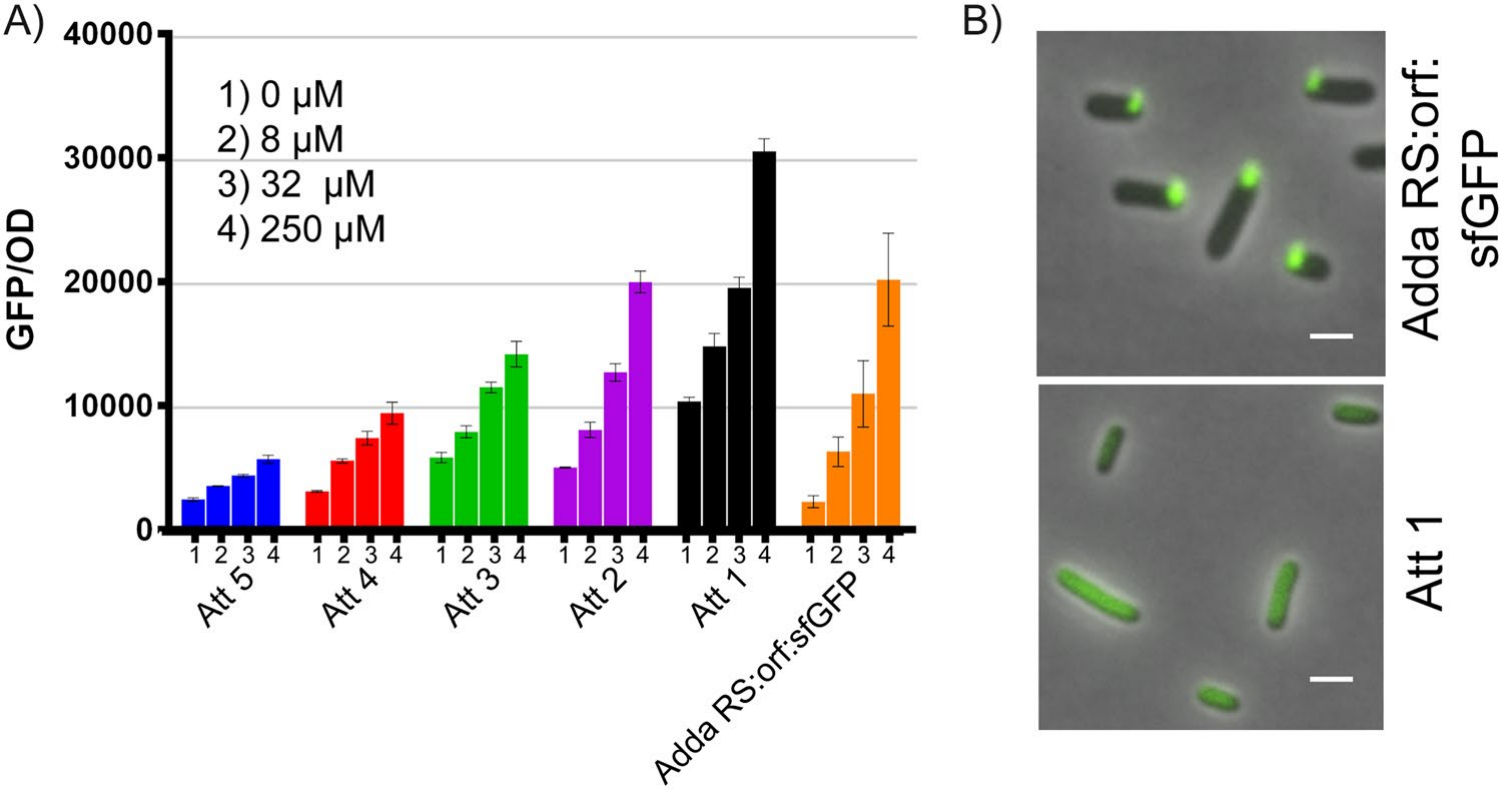
Ribo-attenuators allow addA riboswitch rational re-engineering: (**A**) Response of each ribo-attenuator to 0, 8, 32 and 250 µM 2-aminopurine as compared to the fusion construct (orange). Error bars indicate standard deviation of measurements for biological triplicates. Full single-cell data is presented in Supplementary Fig. 6. (**B**) Fluorescence microscopy of the addA fusion showing clear inclusion bodies and the Att1 ribo-attenuator showing soluble dispersed GFP. White bars represent 1 µm.

**Figure 5:**
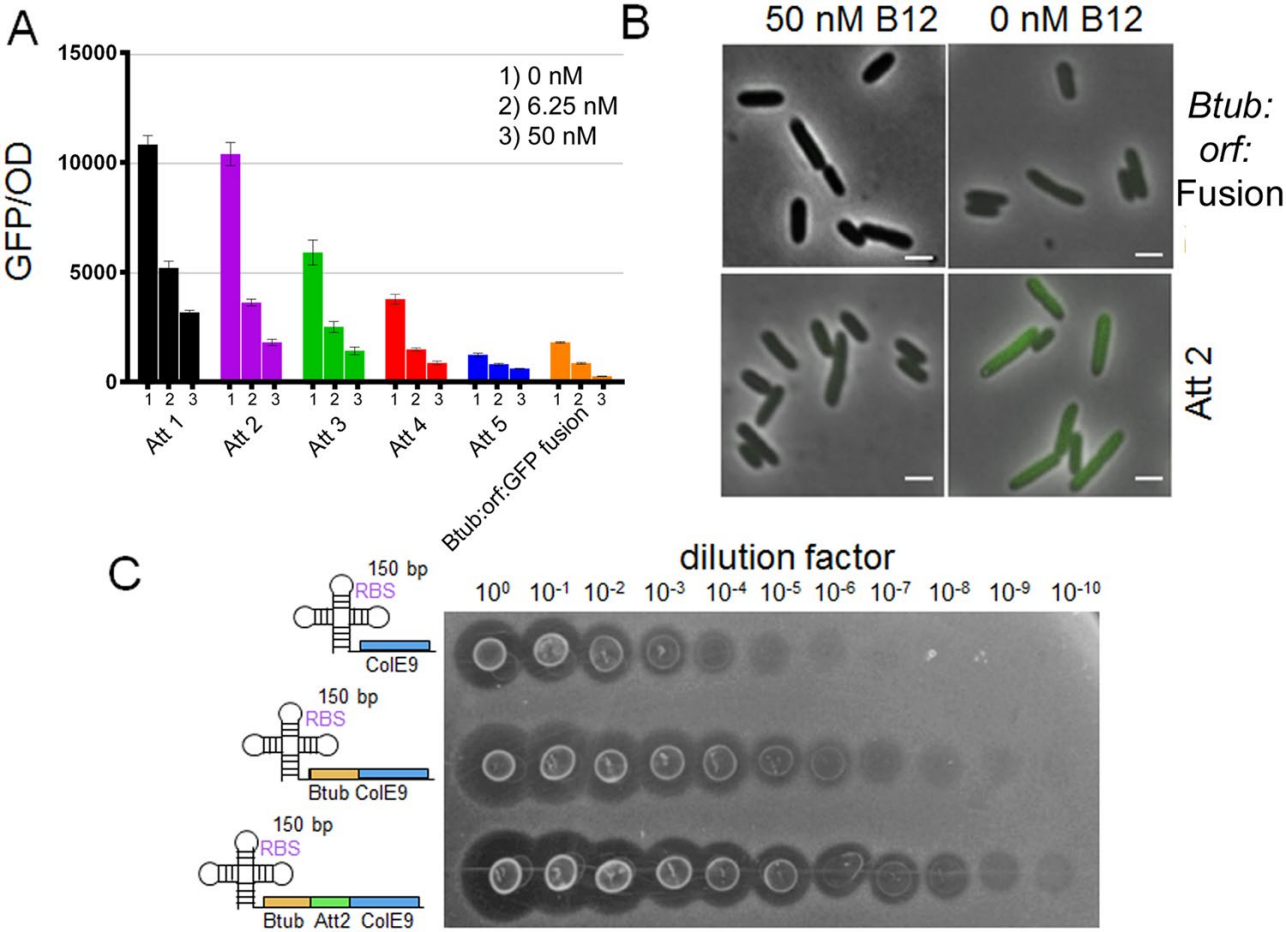
Ribo-attenuators allow btuB riboswitch rational re-engineering: (**A**) Response of each ribo-attenuator to 0, 6.25, and 50 µM 2-aminopurine as compared to the fusion construct (orange). Error bars indicate standard deviation of measurements for biological triplicates. Full single cell data is shown in Supplementary Fig. 6. (**B**) Fluorescence microscopy of the uninduced and induced btuB fusion and Att2 ribo-attenuated btuB riboswitch, with the latter demonstrating a greater induction difference. White bars represent 1 µm. (**C**) Plate-killing assay for production of bacteriocin ColE9 from the btuB riboswitch with and without fusion and attenuator domains. Clearances in the *E*. *coli* soft agar plate correlate with ColE9 production level, demonstrating a substantially increased protein yield in the ribo-attenuator system.

Ribo-attenuators were designed within the addA riboswitch system, placed between 150 bp of the system’s original working ORF (eGFP) and the new gene of interest (sfGFP), as illustrated in Fig. 2. This particular length of ORF (150 bp) was selected by balancing the requirement that it be long enough to include the secondary structure of most riboswitches (which may extend ~100 bp into the open reading frame^17^), but short enough to minimise the overall size of the system. A common RBS region was designed to include the SD1 RBS from the BIOFAB parts library^47^, which was placed upstream of a transcriptionally-coupled junction (TAATG) that included a stop codon in-frame with any ribosome originating from the riboswitch, and a negative one base shifted start codon. Hairpins with ever lower ∆G were introduced over the RBS on the 5′ end by altering the attenuator’s repressing region (Supplementary Fig. 1). For one attenuator (Att2) we took a different approach, engineering a hairpin on the 3′ side by altering bases within the common RBS region. Nine potential hairpins with stems of between 3 and 24 bp in length were screened (Supplementary Fig. 2), from which we selected five (labelled Att1-5) that gave a range of output responses. Further ribo-attenuators could easily be designed rationally around a new RBS by following our process, or by generating random sequences and screening for a ribo-attenuator of the required strength. The selected ribo-attenuators (without upstream riboswitch) were expressed under the control of the tetracycline promoter, demonstrating that with the exception of the smallest hairpin (Att1), the hairpin structures caused a significant drop in the RBS’s translational efficiency (Supplementary Fig. 3). Thus, when a riboswitch is placed upstream of the ribo-attenuator, translation from the attenuator’s RBS independent of riboswitch activity will be minimal. There is some possibility that the expression of this small 150 bp leader peptide could lead to some toxic effects. However, subsequent cell morphology as seen in Figs 4B and 5B was normal and growth rate (not shown) was not impeded by the expression of this leader peptide.

### Ribo-attenuators can shift the addA riboswitch induction response

Five different ribo-attenuators were introduced downstream of the adenine riboswitch and 150 bp of its working ORF (eGFP). The ribo-attenuators were able to shift the system’s induction response to 2-aminopurine in a manner that correlated with the strength of the attenuator; a stronger hairpin leads to a narrower and lower induction response (Fig. 4A). The direct fusion of sfGFP to the working ORF yielded very clear inclusion bodies, manifested as distinct spots present at one pole of the cell (Fig. 4B), which are formed by misfolded insoluble proteins. By comparison, the inclusion of a ribo-attenuator between the riboswitch and sfGFP yielded soluble protein (Att1 shown) since the translated reporter gene is expressed orthogonally from the upstream 150 base pairs of the working ORF. The absence of the fusion domain in the ribo-attenuated case was confirmed via immunoblotting, which demonstrated a size difference between the protein products in systems with and without the attenuator (Supplementary Fig. 4).

To demonstrate the universal applicability of ribo-attenuators, the addA riboswitch was used in isolation, as a 150 bp fusion with its working ORF (eGFP), and with Att2, to express and analyse the targeting of OmpT, an outer membrane protein. For all constructs, basal expression of OmpT was observed (Supplementary Fig. 5). The riboswitch in isolation failed to produce membrane-bound OmpT, whilst in the fusion system OmpT mainly formed insoluble inclusion bodies. However, the ribo-attenuated system yielded correctly targeted OmpT in the cell membrane, demonstrating the ribo-attenuator’s ability to express working protein.

### Ribo-attenuators can shift and amplify btuB riboswitch induction response

The same five ribo-attenuators were introduced downstream of the btuB riboswitch and 150 bp of its working ORF (btuB). The ribo-attenuators were able to shift and substantially amplify the system’s induction profile, with (as for the addA riboswitch) increased hairpin strength corresponding with reduced expression (Fig. 5A). Introduction of the attenuators resulted in a notable difference between the activated and unactivated systems when observed by fluorescence microscopy (Fig. 5B). The use of the same five ribo-attenuators demonstrates the modularity of our system: despite these attenuators being designed for the addA riboswitch system, when transplanted into the btuB system their functionality is maintained.

To demonstrate an additional application of ribo-attenuators, the btuB riboswitch was used in isolation, as a 150 bp fusion with its working ORF (btuB), and with Att2, to express ColE9, a bacteriocin that invades (via OmpF and btuB) and attacks the genome of *E*. *coli* cells^48^, preventing their growth. The amount of active ColE9 in a sample can be directly correlated with the clearance of *E*. *coli* grown on a soft agar plate when ColE9 is spotted onto it^37^. As in Fig. 3B, it was found that inserting ColE9 directly after the riboswitch resulted in minimal expression (Fig. 5C). Expression was increased by a factor of ∼ 1000 in the fusion system, however, with the inclusion of Att2 a substantially higher yield of active protein was achieved. A cleared halo around the cells was observable up to dilution by a factor of 10^10^, demonstrating the attenuator’s ability to significantly enhance protein production from this riboswitch.

### Ribo-attenuators reduce the uncertainty of riboswitch function

**Figure 6:**
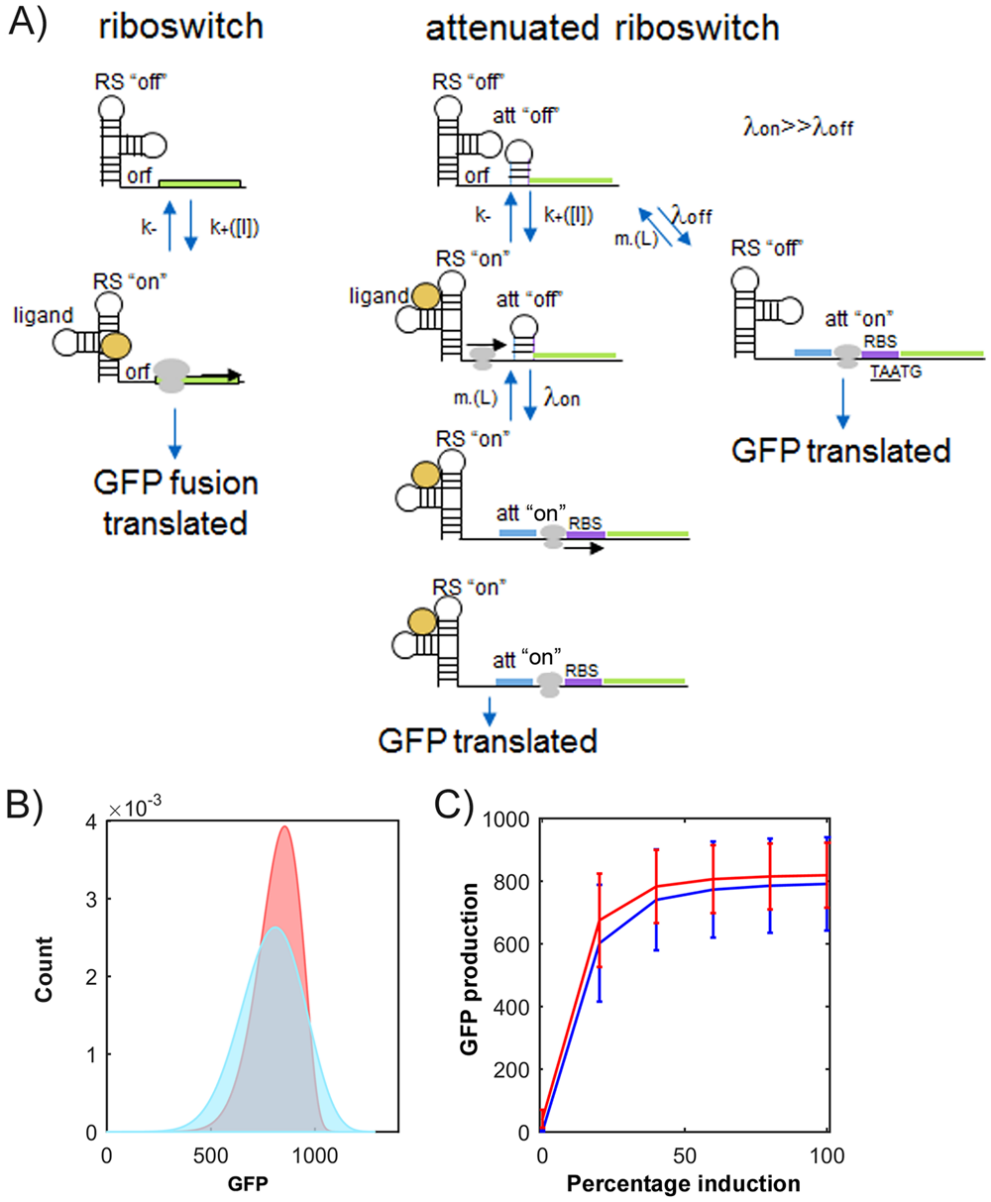
Modelled Ribo-attenuators reduce riboswitch expression noise: (**A**) Random walk dynamics of the modelled system. The riboswitch randomly switches between OFF and ON states at rates k_+_[I] and k_−_, and GFP is produced at rate *λ*_*OFF*_ or *λ*_*ON*_ depending on the state of the riboswitch. In the ribo-attenuator system, the riboswitch has similar dynamics; the rates *λ*_*OFF*_ and *λ*_*ON*_ are now the rates at which the downstream attenuator is switched ON. The downstream attenuator can spontaneously switch o at rate *m*___(*L*), which is an increasing function of the length L of the attenuator’s hairpin stem. GFP is produced at rates µ_*OFF*_ and µ_*ON*_ depending on the state of the downstream attenuator. (**B**) Probability distribution for the random variable defined as the number of GFP molecules expressed by the stochastic model in (**A**) over a unit time interval starting from an arbitrary (long-term) time-point at maximal (100%) induction for the riboswitch system (blue) and the ribo-attenuator system (red). (**C**) The expression distribution mean plus/minus one standard deviation (error bars) for varying induction concentrations defined as a percentage of maximal induction (the data points at 100% induction therefore correspond to the data in (**B**)). Comparison of the riboswitch (blue) and ribo-attenuator system (red) demonstrates substantially reduced variability across the induction range.

The translation of GFP from mRNA in the modelled riboswitch system depends on the state of the riboswitch at any given time, either *λ*_*ON*_ or *λ*_*OFF*_ (Fig. 6A). Over time, the riboswitch stochastically switches between ON and OFF, with inducer concentration biasing the stochastic switching towards one or the other state. Assuming that the two translation rates are at different orders of magnitude, the waiting time from any given time until the production of a GFP molecule is thus highly uncertain. In the modelled ribo-attenuator system, the accessibility of the two RBSs can be in one of four states (OFF, OFF), (OFF, ON), (ON, OFF), and (ON, ON) (Fig. 6A). The rates *λ*_*ON*_ and *λ*_*OFF*_ of ribosome transit now determine the rates at which the second switch turns on, given the state of the first. The scale separation of those rates means that, to a first order approximation, the first switch ON implies the second switch will turn ON, and the first switch OFF implies the second switch is likely to turn OFF. However, the GFP translation rates µ_*ON*_ and µ_*OFF*_ resulting from the state of the second switch can now be set independently of the translation parameters of the upstream riboswitch via adjustment of the ribo-attenuator’s RBS. Expression level is also dependent upon the rate at which the ribo-attenuator switches back to the OFF position (*m*___(*L*)) which is itself a function of the length (L) and hence strength of the attenuator region’s secondary structure.

**Figure 7:**
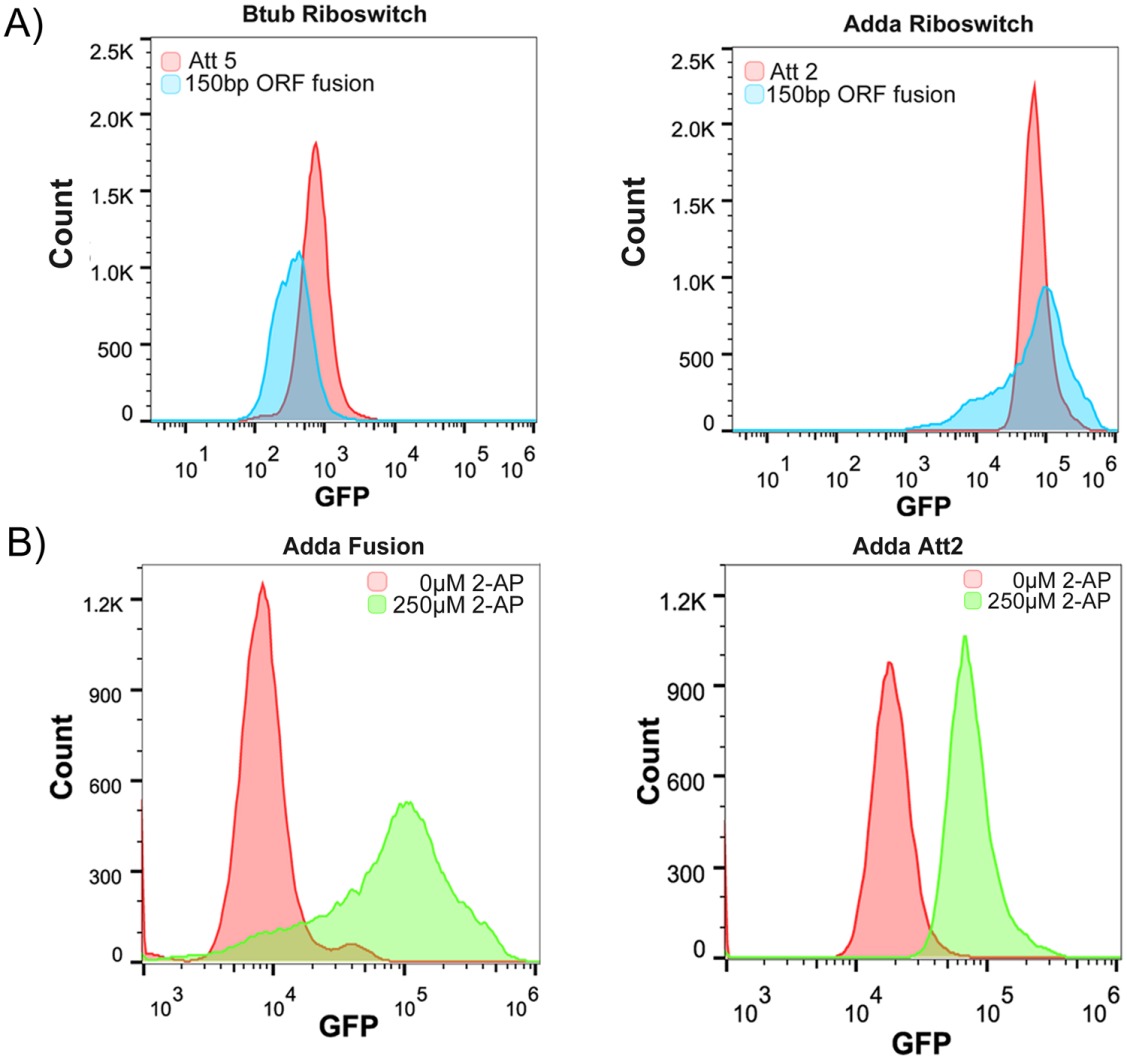
Measured ribo-attenuators reduce riboswitch expression noise: (**A**) Measured expression distributions at maximal induction for the btuB riboswitch with a fusion system (blue) and with Att5 (red), and for the addA riboswitch with a fusion system (blue) and Att2 (red). (**B**) Single cell expression distributions at the lower and upper limits of the addA riboswitch system’s induction with and without inclusion of the Att2 ribo-attenuator. Equivalent data for the btuB system is presented in Supplementary Fig. 7.

From this stochastic model, probability distributions for total GFP expression were generated, demonstrating a substantial reduction in gene expression variability at maximal induction for the ribo-attenuator system when compared to the un-attenuated riboswitch (Fig. 6B), a trend which is maintained over a range of induction levels (Fig. 6C). The same behaviour was observed experimentally for both the addA and btuB riboswitches (Fig. 7A), which demonstrates that inclusion of a ribo-attenuator substantially reduces the width of the expression distribution. Figure 7A displays the single cell distinction between uninduced and fully induced populations for the addA riboswitch with and without ribo-attenuator, demonstrating that the reduced variation in the attenuated system makes overlapping populations substantially more distinct. A similar trend was observed for the btuB riboswitch (Supplementary Fig. 7).

## Discussion

We designed a novel and widely applicable tool that allows riboswitches to be used as modular, tunable components with a highly predictable outcome, enhancing applicability by overcoming previously identified drawbacks. We chose two well known, representative riboswitches; the addA riboswitch, an activating riboswitch that respond to a cellular metabolite (adenine), and the btuB riboswitch, a repressing riboswitch which responds to adenosylcobalamin. These have the potential to facilitate the biological production of high value micronutrients such as vitamins, many of which are still produced via expensive and inefficient chemical synthesis. Recently described methods applying riboswitches to high value vitamins could also be used with the ribo-attenuators described herein to elicit a high throughput approach to pathway elucidation^49^.

The first advantage of the developed ribo-attenuator system is that it enables riboswitch controlled expression of a new gene of interest without the inclusion of a 5′ fusion. Typically such fusions have been employed^18–21^ because direct replacement of a riboswitch’s ORF can degrade its functionality^17^ (as demonstrated in Fig. 3). However, re-engineering a riboswitch in this fashion gives rise to a protein with a large fusion, which can severely impact function. This was demonstrated in Fig. 4B, where the fusion-based approach led to formation of inclusion bodies. As a second example, expression of the outer membrane protein OmpT demonstrated that fusion to the first 150 bp of btuB resulted in formation of inclusion bodies, whereas the attenuator-produced protein was correctly targeted to the membrane. We hypothesise that inclusion bodies may form due to the fusion domain causing protein mis-folding. Alternatively, in the case of OmpT the fusion domain’s presence may disrupt the signal sequence required for membrane transport^50^. Ribo-attenuators overcome this challenge by translating a gene of interest from a second downstream RBS, avoiding inclusion of any 5′ fusion and thereby improving functionality.

A second benefit provided by the inclusion of a ribo-attenuator is the ability to modularly tune the induction response of a riboswitch. This was demonstrated by ribo-attenuators shifting and re-scaling the output response of the addA system in which they were designed (Fig. 4A), in a manner correlating with *a priori* predictions of their relative strength. This functionality was maintained when the same ribo-attenuators were employed in a different genetic context in the btuB system (Fig. 5A), demonstrating their modularity. In both contexts, an increased expression level was observed (compared to the fusion system) when the riboswitch was in its repressing induction state. This may arise due to spontaneous accessibility of the ribo-attenuator’s RBS, or re-initiation of terminated ribosomes at the ribo-attenuator’s start codon due to 70 s scanning^51^. Furthermore, an increase in repressed expression level could occur due to the fact that any ribosome originating from the riboswitch RBS would, in the fusion case, produce a single copy of GFP, whilst in the ribo-attenuator system it would open the attenuator’s hairpin for a time period during which multiple translation initiation may events occur. Particularly in the btuB system, ribo-attenuators were able to substantially increase the maximal induction output (for both GFP and ColE9). This is an important capability^31^ that would be difficult to achieve via re-engineering of the riboswitch’s RBS (which could in influence its ligand-binding ability), or via increasing translational promoter strength (our system already employed the strong tetracycline promoter at full induction). Though attempts have been made to overcome these problems, such as boosting riboswitch efficiency through RNA amplification^31^ or *de novo* design of synthetic riboswitches^30^, as-yet they do not provide a reliable, modular, and predictable approach to tuning of riboswitch induction. Our ribo-attenuator system achieves this, and new variants can be developed with different RBS strengths and easily predicted secondary structures, whilst preserving riboswitch ligand response. It is however possible that a completely new genetic context could cause a ribo-attenuator to act unreliably through the introduction of new secondary structure not predicted. Although we did not observe this with these ribo-attenuators, further *in vitro* modelling of RNA secondary structures could mitigate any issues that arise and a new, functional ribo-attenuator could easily be designed for a new genetic context.

A third advantage provided by ribo-attenuators is their ability to reduce variability of a riboswitch’s output. This limitation had restricted the application of riboswitches in a number of ways: For example, unpredictable behaviour may produce downstream variance in processes under a riboswitch’s control, whilst also making separations of populations by FACS challenging, limiting the ability to screen libraries for novel compounds. Our modelled ribo-attenuator system provided a theoretical underpinning for its noise-reduction capability, demonstrating that inclusion of a ribo-attenuator can cause a substantial reduction in population expression variability, as well as a slight increase in mean expression (Fig. 6B). This was observed experimentally (Fig. 7A), demonstrating that the reduction in variability can provide more distinct output distributions (Fig. 7B).

In conclusion, by fine-tuning sensitivity and reducing uncertainty in expression, ribo-attenuators facilitate the use of riboswitches in applications ranging from FACS screening of component libraries to rapid elucidation of novel biosynthesis pathways. Initially we examined the sensitivity of two well-known riboswitches to the introduction of a new open reading frame: Introducing a novel reporter as part of a fusion yielded functional riboswitches, but inclusion bodies that significantly limit application were observed. We also found a high degree of variation in the addA riboswitch, which made identification of specific concentrations of ligand difficult. Using ribo-attenuators we allowed both riboswitches to maintain the original open reading frame and as a result their ligand response, whist expressing the introduced gene of interest orthogonally over a predictable dynamic range. Using these ribo-attenuators it should be possible to overcome limitations to riboswitches identified in previous studies. For example, the glycine riboswitch is a similar riboswitch from *Bacillus subtilis* identified as having an unsuitable induction range^52^, which limits its use as a tool for maintaining vectors in the absence of an antibiotic, or as a cheap glycine-based induction system for bioreactors. As shown in Fig. 5 for the btuB riboswitch, ribo-attenuators could amplify and tune this system’s static response, aiding its industrial application. In summary, ribo-attenuators represent a breakthrough step towards allowing riboswitches to be treated as truly modular and tunable devices.

## Electronic supplementary material

Supplementary Information

## References

[1] Serganov A, Patel DJ (2007). Ribozymes, riboswitches and beyond: regulation of gene expression without proteins. Nat. Rev. Gen.

[2] Groisman EA, Cromie MJ, Shi Y, Latifi T (2006). A Mg2_+_-responding RNA that controls the expression of a Mg2_+_ transporter. Cold SH. Q. B.

[3] Mandal M, Breaker RR (2004). Adenine riboswitches and gene activation by disruption of a transcription terminator. *Nat*. *Struct*. &. Mol. Biol.

[4] Serganov A, Huang L, Patel DJ (2008). Structural insights into amino acid binding and gene control by a lysine riboswitch. Nature.

[5] Wacker A (2011). Structure and dynamics of the deoxyguanosine-sensing riboswitch studied by NMR-spectroscopy. Nucleic Acids Res.

[6] Li S, Breaker RR (2013). Eukaryotic TPP riboswitch regulation of alternative splicing involving long-distance base pairing. Nucleic Acids Res.

[7] Rinaldi AJ, Lund PE, Blanco MR, Walter NG (2016). The Shine-Dalgarno sequence of riboswitch-regulated single mRNAs shows ligand-dependent accessibility bursts. Nature Commun.

[8] Reining A (2013). Three-state mechanism couples ligand and temperature sensing in riboswitches. Nature.

[9] Chen YY, Galloway KE, Smolke CD (2012). Synthetic biology: advancing biological frontiers by building synthetic systems. Genome Biol..

[10] Breaker, R. R. Riboswitches and the RNA world. *CSH*. *Perspect*. *Biol*. **4** (2012). 10.1101/cshperspect.a003566 PMC3281570 21106649

[11] Isaacs FJ (2004). Engineered riboregulators enable post-transcriptional control of gene expression. Nat. Biotechnol..

[12] Rodrigo G, Landrain TE, Majer E, Daros JA, Jaramillo A (2013). Full Design Automation of Multi-State RNA Devices to Program Gene Expression Using Energy-Based Optimization. PLoS Comput. Biol..

[13] Wittmann A, Suess B (2011). Selection of tetracycline inducible self-cleaving ribozymes as synthetic devices for gene regulation in yeast. Mol. BioSyst..

[14] Wieland M, Hartig JS (2008). Artificial Riboswitches: Synthetic mRNA-Based Regulators of Gene Expression. ChemBioChem.

[15] Dixon N (2010). Reengineering orthogonally selective riboswitches. PNAS.

[16] Lin, M. T. *et al*. Novel Utilization of Terminators in the Design of Biologically Adjustable Synthetic Filters. *ACS Syn*. *Bio*. **5** (2016). 10.1021/acssynbio.5b00174 26912179

[17] Caron MP (2012). Dual-acting riboswitch control of translation initiation and mRNA decay. PNAS.

[18] Franklund CV, Kadner RJ (1997). Multiple transcribed elements control expression of the *Escherichia coli* btuB gene. J. Bacteriol.

[19] Nou X, Kadner R (1998). J. Coupled changes in translation and transcription during cobalamin-dependent regulation of btuB expression in. Escherichia coli. J. Bacteriol..

[20] Winkler W, Nahvi A, Breaker RR (2002). Thiamine derivatives bind messenger RNAs directly to regulate bacterial gene expression. Nature.

[21] Dixon N (2012). Orthogonal riboswitches for tuneable coexpression in bacteria. Angew. Chem. Int. Edit.

[22] Renzi F (2006). Large-scale purification and crystallization of the endoribonuclease XendoU: troubleshooting with His-tagged proteins. Acta Crystallogr. F.

[23] Amor-Mahjoub M, Suppini JP, Gomez-Vrielyunck N, Ladjimi M (2006). The effect of the hexahistidine-tag in the oligomerization of HSC70 constructs. J. Chromatogr. B..

[24] Chant A, Kraemer-Pecore CM, Watkin R, Kneale GG (2005). Attachment of a histidine tag to the minimal zinc finger protein of the *Aspergillus nidulans* gene regulatory protein AreA causes a conformational change at the DNA-binding site. Protein Expres. Purif.

[25] Woestenenk EA, Hammarström M, van den Berg S, Härd T, Berglund H (2004). His tag effect on solubility of human proteins produced in *Escherichia coli:* a comparison between four expression vectors. J. Struct. Funct. Genomics.

[26] Wu J, Filutowicz M (1999). Hexahistidine (His6)-tag dependent protein dimerization: a cautionary tale. Acta Biochimica. Pol.

[27] Horchani H, Ouertani S, Gargouri Y, Sayari A (2009). The N-terminal His-tag and the recombination process affect the biochemical properties of Staphylococcus aureus lipase produced in *Escherichia coli*. J. Mol. Catal. B-Enzym..

[28] Wachsmuth M, Findeiß S, Weissheimer N, Stadler PF, Mörl M (2013). De novo design of a synthetic riboswitch that regulates transcription termination. Nucleic Acids Res.

[29] Beisel CL, Smolke CD (2009). Design Principles for Riboswitch Function. PLoS Comp. Biol.

[30] Espah Borujeni A, Mishler DM, Wang J, Huso W, Salis HM (2016). Automated physics-based design of synthetic riboswitches from diverse RNA aptamers. Nucleic Acids Res.

[31] Emadpour M, Karcher D, Bock R (2015). Boosting riboswitch efficiency by RNA amplification. Nucleic Acids Res.

[32] Bonnet J, Yin P, Ortiz ME, Subsoontorn P, Endy D (2013). Amplifying genetic logic gates. Science.

[33] Gibson DG (2009). Enzymatic assembly of DNA molecules up to several hundred kilobases. Nat Methods.

[34] Gibson DG (2010). Creation of a bacterial cell controlled by a chemically synthesized genome. Science.

[35] Lutz R, Bujard H (1997). Independent and tight regulation of transcriptional units in *Escherichia coli* via the LacR/O, the TetR/O and AraC/I1-I2 regulatory elements. Nucleic acids Res.

[36] Folliard, T. Ribo-attenuators: novel elements for reliable and modular riboswitch engineering. *DATA*, doi:10.5287/bodleian:mNNpdYbpN (2017). 10.1038/s41598-017-04093-x PMC5496857 28676696

[37] Ridley H, Lakey JH (2015). Antibacterial toxin colicin N and phage protein G3p compete with TolB for a binding site on TolA. Microbiol.

[38] Mertins, B. *et al*. Flexibility of the N-Terminal mVDAC1 Segment Controls the Channel’s Gating Behavior. *PLoS ONE* **7** (2012). 10.1371/journal.pone.0047938 PMC3479125 23110136

[39] Laemmli UK (1970). Cleavage of Structural Proteins during Assembly of Head of Bacteriophage-T4. Nature.

[40] Towbin H, Staehelin T, Gordon J (1979). Electrophoretic transfer of proteins from polyacrylamide gels to nitrocellulose sheets: procedure and some applications. PNAS.

[41] Serganov A (2004). Structural basis for discriminative regulation of gene expression by adenine and guanine-sensing mRNAs. Chem. Biol..

[42] Gong S, Wang Y, Zhang W (2015). Kinetic regulation mechanism of pbuE riboswitch. J. Chem. Phys..

[43] Rieder R, Lang K, Graber D, Micura R (2007). Ligand-Induced Folding of the Adenosine Deaminase A-Riboswitch and Implications on Riboswitch Translational Control. ChemBioChem.

[44] Gilbert SD, Montange RK, Stoddard CD, Batey RT (2006). Structural studies of the purine and SAM binding riboswitches. Cold. SH. Q. B.

[45] Cotlet M, Goodwin PM, Waldo GS, Werner JH (2006). A Comparison of the Fluorescence Dynamics of Single Molecules of a Green Fluorescent Protein: One-versus Two-Photon Excitation. ChemPhysChem.

[46] Zhang G, Gurtu V, Kain SR (1996). An enhanced green fluorescent protein allows sensitive detection of gene transfer in mammalian cells. Biochem. Bioph. Res. Co..

[47] Mutalik VK (2013). Precise and reliable gene expression via standard transcription and translation initiation elements. Nat. Methods.

[48] Kleanthous C (2010). Swimming against the tide: progress and challenges in our understanding of colicin translocation. Nature Rev. Microbiol..

[49] Zhu X, Wang X, Zhang C, Wang X, Gu Q (2015). A riboswitch sensor to determine vitamin B12 in fermented foods. Food Chem..

[50] Kumamoto CA, Oliver DB, Beckwith J (1984). Signal sequence mutations disrupt feedback between secretion of an exported protein and its synthesis in *E*. *coli*. Nature.

[51] Yamamoto H (2016). 70S-scanning initiation is a novel and frequent initiation mode of ribosomal translation in bacteria. PNAS.

[52] Phan TTP, Schumann W (2007). Development of a glycine-inducible expression system for Bacillus subtilis. J. Biotechnol..

[53] Newport, T. Ribo-attenuator animation. *Figshare*, doi:10.6084/m9.figshare.4587061.v1 (2017).

