# Supplementary material for: Ribo-attenuators: novel elements for reliable and modular riboswitch engineering
Source: Sci Rep. 2017 Jul 4;7:4599. doi: 10.1038/s41598-017-04093-x (PMC5496857; doi:10.1038/s41598-017-04093-x)
Supplement: Supplementary file 1 — Supplementary Information [file 41598_2017_4093_MOESM1_ESM.pdf]

Supplementary Information, Ribo-attenuators: novel elements for reliable and modular riboswitch engineering

Thomas Folliard*†[1], Barbara Mertins*[1], Harrison Steel[2], Thomas P Prescott[2], Thomas Newport[1], Christopher W Jones[1], George Wadhams[1], Travis Bayer[3], Judith P Armitage[1], Antonis Papachristodoulou[2] and Lynn J Rothschild‡[4]

[1]Department of Biochemistry, University of Oxford, South Parks Road, Oxford OX1 3PJ, UK
[2]Department of Engineering Science, University of Oxford, Parks Road, Oxford OX1 3PJ, UK
[3]Asimolar Bio, inc., 953 Indiana St, San Francisco, CA 94107, USA
[4]National Aeronautics and Space Administration Ames Research Center, Moffett Field, CA 94035, USA

1 Models

We consider two models: the one-component system (a riboswitch), and the two-component system (a riboswitch with attached ribo-attenuator).

1.1 One-Component System

Here the RNA has a single riboswitch covering the RBS for GFP. There are two possible rates at which ribosomes bind to the RNA, depending on the state of the riboswitch covering the RBS. If the riboswitch is in the OFF position, GFP is translated at a rate λ_{OFF}. If the riboswitch is in the ON position, the translation rate of GFP is $\lambda_{ON} \gg \lambda_{OFF}$. GFP also degrades at a constant rate δ. The riboswitch randomly flips between OFF and ON, depending on the inducer concentration $[I]$, such that

$$OFF \underset{k_-}{\overset{k_+([I])}{\rightleftharpoons}} ON, \tag{1}$$

where $k_+([I])$ is assumed to be an increasing function of $[I]$. This is a random walk, where increasing inducer concentrations correspond to a bias towards the ON state.

1.2 Two-Component System

Here there are two switches on the RNA: the original riboswitch, and the attenuator. There are four possible states of the two switches: (OFF, OFF), (OFF, ON), (ON, OFF), and (ON, ON). In each of these states, the first ON or OFF corresponds to the state of the first switch, while the second ON or OFF corresponds to the state of the second switch. The intuition behind this system is that a ribosome binds to the RBS downstream of the first switch at the rate λ_{OFF} or λ_{ON} defined above, depending on its state. The ribosome then opens up the downstream switch. Ribosomes also bind to another RBS downstream of the first, and translate GFP at two rates μ_{OFF} and $\mu_{ON} \gg \mu_{OFF}$, now depending on the state of the second switch. Again, GFP degrades at a rate δ.

*These authors contributed equally to this work
†e-mail: thomas.folliard@oriel.ox.ac.uk
‡e-mail: Lynn.J.Rothschild@nasa.gov

The possible transitions between the four states of the two switches are:

$$(OFF, OFF) \underset{k_-}{\overset{k_+([I])}{\rightleftharpoons}} (ON, OFF), \tag{2a}$$

$$(OFF, OFF) \underset{m_-(L)}{\overset{\lambda_{OFF}}{\rightleftharpoons}} (OFF, ON), \tag{2b}$$

$$(ON, OFF) \underset{m_-(L)}{\overset{\lambda_{ON}}{\rightleftharpoons}} (ON, ON), \tag{2c}$$

$$(OFF, ON) \underset{k_-}{\overset{k_+([I])}{\rightleftharpoons}} (ON, ON). \tag{2d}$$

Here $k_\pm$ are as in the previous model, describing the dynamics of the upstream switch. There are two rates that the downstream region switches on: λ_{OFF} and λ_{ON}, depending on the upstream switch's state. The downstream switch spontaneously switches off at a rate $m_-(L)$ that we assume is an increasing function of L, the length of the attenuator region.

To emphasise: the two-component system has production rates of GFP of μ_{OFF} in the states (OFF, OFF) and (ON, OFF), and $\mu_{ON} \gg \mu_{OFF}$ in the states (OFF, ON) and (ON, ON).

2 Analysis

Our analysis of these random processes focused on a single random variable. We defined T to be the length of time elapsing from an arbitrary time τ until the next GFP molecule was produced. We assumed that both systems had reached stationarity, so that this was well-defined independently of τ. Importantly, this random variable is *not* equal to the time elapsed between two consecutive GFP production events: instead, the start time τ is arbitrary. In the main text, the inverse $1/T$ of this random variable is what we call the expression rate.

2.1 One-Component System

To find the CDF of T, we condition on the state of the RNA switch, so that

$$\mathbb{P}(T \leq t) = \mathbb{P}(T \leq t \mid OFF)\mathbb{P}(OFF) + \mathbb{P}(T \leq t \mid ON)\mathbb{P}(ON).$$

It is simple to see that, at stationarity, we have $\mathbb{P}(ON) = k_+/(k_+ + k_-)$ and $\mathbb{P}(OFF) = k_-/(k_+ + k_-)$, where the notation of $[I]$ is dropped for now. We define two conditional CDFs $F_{OFF}(t) := \mathbb{P}(T \leq t \mid OFF)$ and $F_{ON}(t) := \mathbb{P}(T \leq t \mid ON)$, with boundary conditions $F_{OFF}(0) = F_{ON}(0) = 0$ and $\lim_{t\to\infty} F_{OFF}(t) = \lim_{t\to\infty} F_{ON}(t) = 1$.

Assume that the switch at time t is ON, and consider the auxiliary random variable X, which is the waiting time until the switch switches OFF. Conditioning on $X \geq 0$, the conditional CDF $F_{ON}(t)$ satisfies the equation

$$\begin{aligned}
F_{ON}(t) = \mathbb{P}(T \leq t \mid ON) &= \int_0^\infty \mathbb{P}(T \leq t \mid ON, X = x) k_- e^{-k_- x} \, \mathrm{d}x \\
&= \int_t^\infty \mathbb{P}(T \leq t \mid ON, X = x) k_- e^{-k_- x} \, \mathrm{d}x + \int_0^t \mathbb{P}(T \leq t \mid ON, X = x) k_- e^{-k_- x} \, \mathrm{d}x \\
&= \left(1 - e^{-\lambda_{ON} t}\right) e^{-k_- t} + \int_0^t \left[\left(1 - e^{-\lambda_{ON} x}\right) + e^{-\lambda_{ON} x} F_{OFF}(t - x)\right] k_- e^{-k_- x} \, \mathrm{d}x \\
&= \frac{\lambda_{ON}}{\lambda_{ON} + k_-}\left(1 - e^{-(\lambda_{ON} + k_-)t}\right) + e^{-(\lambda_{ON} + k_-)t} \int_0^t F_{OFF}(s) k_- e^{(\lambda_{ON} + k_-)s} \, \mathrm{d}s.
\end{aligned}$$

We multiply this equation by $e^{(\lambda_{ON}+k_-)t}$, and take the derivative of both sides with respect to t. Re-arranging the result, we find that the PDF $f_{ON}(t) = \frac{\mathrm{d}}{\mathrm{d}t} F_{ON}(t)$ is equal to

$$f_{ON}(t) = -(\lambda_{ON} + k_-)F_{ON}(t) + k_- F_{OFF}(t) + \lambda_{ON}. \tag{3a}$$

By the symmetry of our notation (or a similar argument), the PDF of T conditional on the OFF switch state satisfies

$$f_{OFF}(t) = -(\lambda_{OFF} + k_+)F_{OFF}(t) + k_+ F_{ON}(t) + \lambda_{OFF}. \tag{3b}$$

Hence, matrix notation gives a first-order, two-dimensional ODE to solve for F_{ON} and F_{OFF}:

$$\begin{bmatrix} f_{ON} \\ f_{OFF} \end{bmatrix} = \begin{bmatrix} -(\lambda_{ON} + k_-) & k_- \\ k_+ & -(\lambda_{OFF} + k_+) \end{bmatrix} \begin{bmatrix} F_{ON} \\ F_{OFF} \end{bmatrix} + \begin{bmatrix} \lambda_{ON} \\ \lambda_{OFF} \end{bmatrix}. \tag{4}$$

Denote the two negative eigenvalues of the system matrix as $-\omega_1$ and $-\omega_2$, with corresponding eigenvectors v_1 and v_2. The solution to this ODE that satisfies the boundary conditions is

$$\begin{bmatrix} F_{ON}(t) \\ F_{OFF}(t) \end{bmatrix} = \begin{bmatrix} 1 \\ 1 \end{bmatrix} - \alpha_1 v_1 e^{-\omega_1 t} - \alpha_2 v_2 e^{-\omega_2 t},$$

where α_1 and α_2 are determined by the eigenvectors such that

$$\alpha_1 v_1 + \alpha_2 v_2 = \begin{bmatrix} 1 \\ 1 \end{bmatrix}.$$

Finally, the CDF $F(t) = \mathbb{P}(T \leq t)$ is equal to $F(t) = 1 - A_1 e^{-\omega_1 t} - A_2 e^{-\omega_2 t}$, for the scalar coefficients

$$A_i = \alpha_i \begin{bmatrix} \frac{k_+}{k_+ + k_-} & \frac{k_-}{k_+ + k_-} \end{bmatrix} v_i.$$

From this CDF we can calculate quantiles, and also determine the mean of T as

$$\mathbb{E}(T) = \frac{A_1}{\omega_1} + \frac{A_2}{\omega_2}.$$

2.2 Two-Component System

We can now perform a similar analysis on the two-component system, but now need to consider the CDF of T conditional on the four possible states of the RNA. Following the example above, we write

$$\begin{aligned} F(t) &= \mathbb{P}(T \leq t) \\ &= \mathbb{P}(T \leq t \mid (OFF, OFF))\mathbb{P}(OFF, OFF) + \mathbb{P}(T \leq t \mid (OFF, ON))\mathbb{P}(OFF, ON) \\ &\quad + \mathbb{P}(T \leq t \mid (ON, OFF))\mathbb{P}(ON, OFF) + \mathbb{P}(T \leq t \mid (ON, ON))\mathbb{P}(ON, ON). \end{aligned}$$

We write the conditional CDFs as $F_{(OFF,OFF)}(t) := \mathbb{P}(T \leq t \mid (OFF, OFF))$ and so on. Given that the RNA is in state (OFF, OFF) at time t, we now let the auxiliary random variable X denote the time elapsing between the arbitrary time t and a switch away from (OFF, OFF). The first conditional CDF $F_{(OFF,OFF)}$ then satisfies the equation

$$\begin{aligned} F_{(OFF,OFF)}(t) &= \mathbb{P}(T \leq t \mid (OFF, OFF)) \\ &= \int_0^\infty \mathbb{P}(T \leq t \mid (OFF, OFF), X = x)(k_+ + \lambda_{OFF})e^{-(k_+ + \lambda_{OFF})x}\, \mathrm{d}x \\ &= \left(1 - e^{-\mu_{OFF} t}\right) e^{-(k_+ + \lambda_{OFF})t} + \int_0^t (1 - e^{-\mu_{OFF} x})(k_+ + \lambda_{OFF})e^{-(k_+ + \lambda_{OFF})x}\, \mathrm{d}x \\ &\quad + \int_0^t \left[e^{-\mu_{OFF} x}\left(k_+ F_{(ON,OFF)}(t - x) + \lambda_{OFF} F_{(OFF,ON)}(t - x)\right)\right] e^{-(k_+ + \lambda_{OFF})x}\, \mathrm{d}x \\ &= \frac{\mu_{OFF}}{\mu_{OFF} + k_+ + \lambda_{OFF}} \left(1 - e^{-(\mu_{OFF} + k_+ + \lambda_{OFF})t}\right) \\ &\quad + e^{-(\mu_{OFF} + k_+ + \lambda_{OFF})t} \int_0^t \left[k_+ F_{ON,OFF}(s) + \lambda_{OFF} F_{OFF,ON}(s)\right] e^{(\mu_{OFF} + k_+ + \lambda_{OFF})s}\, \mathrm{d}s. \end{aligned}$$

We then multiply both sides by $e^{(\mu_{OFF} + k_+ + \lambda_{OFF})t}$ and take derivatives. Re-arranging for the PDF $f_{(OFF,OFF)}$, we find that

$$f_{(OFF,OFF)}(t) = \mu_{OFF} + \lambda_{OFF} F_{(OFF,ON)}(t) + k_+ F_{(ON,OFF)}(t) - (mu_{OFF} + k_+ + \lambda_{OFF})F_{(OFF,OFF)}(t).$$

Performing a similar analysis for the other CDFs, we find that

$$\begin{bmatrix} f_{(OFF,OFF)}(t) \\ f_{(OFF,ON)}(t) \\ f_{(ON,OFF)}(t) \\ f_{(ON,ON)}(t) \end{bmatrix} = \begin{bmatrix} -(\mu_{OFF} + k_+ + \lambda_{OFF}) & \lambda_{OFF} & k_+ & 0 \\ m_- & -(\mu_{ON} + m_- + k_+) & 0 & k_+ \\ k_- & 0 & -(\mu_{OFF} + k_- + \lambda_{ON}) & \lambda_{ON} \\ 0 & k_- & m_- & -(\mu_{ON} + m_- + k_-) \end{bmatrix} \begin{bmatrix} F_{(OFF,OFF)}(t) \\ F_{(OFF,ON)}(t) \\ F_{(ON,OFF)}(t) \\ F_{(ON,ON)}(t) \end{bmatrix} + \begin{bmatrix} \mu_{OFF} \\ \mu_{ON} \\ \mu_{OFF} \\ \mu_{ON} \end{bmatrix}.$$

This is now a four-dimensional first-order ODE, but the analysis mirrors that of the simpler case above. If $-\phi_i$ and w_i, for $i = 1, \ldots, 4$, are the negative eigenvalues and corresponding eigenvectors of this system matrix, then the solution of this ODE satisfying the boundary conditions is

$$\begin{bmatrix} F_{(OFF,OFF)}(t) \\ F_{(OFF,ON)}(t) \\ F_{(ON,OFF)}(t) \\ F_{(ON,ON)}(t) \end{bmatrix} = \begin{bmatrix} 1 \\ 1 \\ 1 \\ 1 \end{bmatrix} - \sum_{i=1}^4 \beta_i w_i e^{-\phi_i t},$$

where the coefficients β_i are chosen such that $\sum_{i=1}^{4} \beta_i w_i = \begin{bmatrix} 1 & 1 & 1 & 1 \end{bmatrix}^T$. These conditional CDFs map to the unconditional CDF for T to give

$$F(t) = \mathbb{P}(T \leq t) = 1 - \sum_{i=1}^{4} B_i e^{-\phi_i t},$$

where

$$B_i = \beta_i \begin{bmatrix} \mathbb{P}(OFF, OFF) & \mathbb{P}(OFF, ON) & \mathbb{P}(ON, OFF) & \mathbb{P}(ON, ON) \end{bmatrix} w_i$$

for the probabilities of the stationary distribution of the random walk with transitions given in the model (2) above. Again, T has a mean

$$\mathbb{E}(T) = \sum_{i=1}^{4} \frac{B_i}{\phi_i}.$$

3 Supplementary data

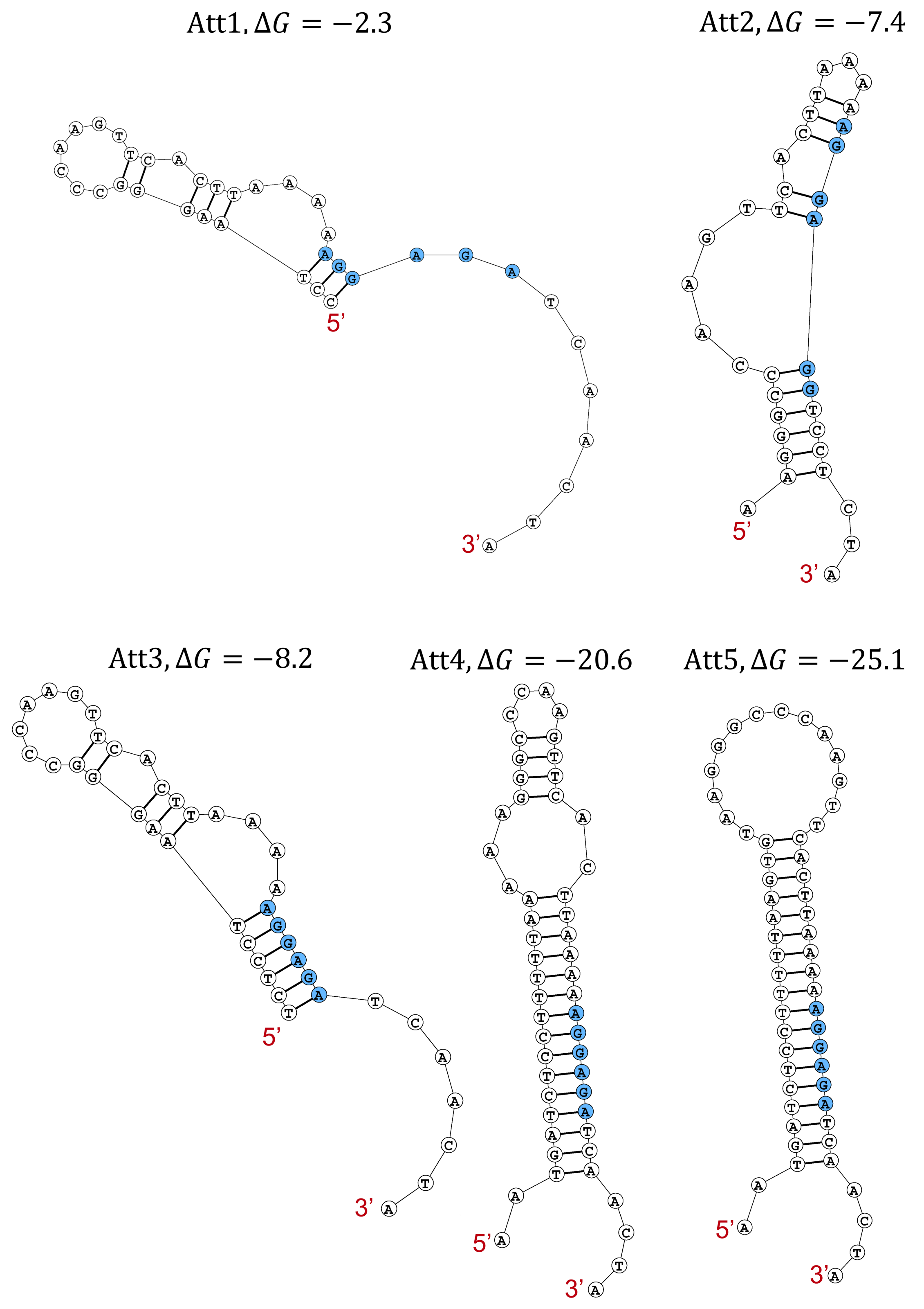

Figure S1: **Ribo-attenuator secondary structures:** Lowest free energy conformations were calculated for each ribo-attenuator using the RNAstructure Fold Web Server[2]. These structures were calculated for the attenuators in isolation, and thus may vary depending on corresponding genetic contexts (Fig. 2), as well as due to limitations inherent in the theoretical prediction of RNA structures. ΔG values are in kcal/mole, and the RBS core is highlighted in blue. For isolated sequences see Table S2.

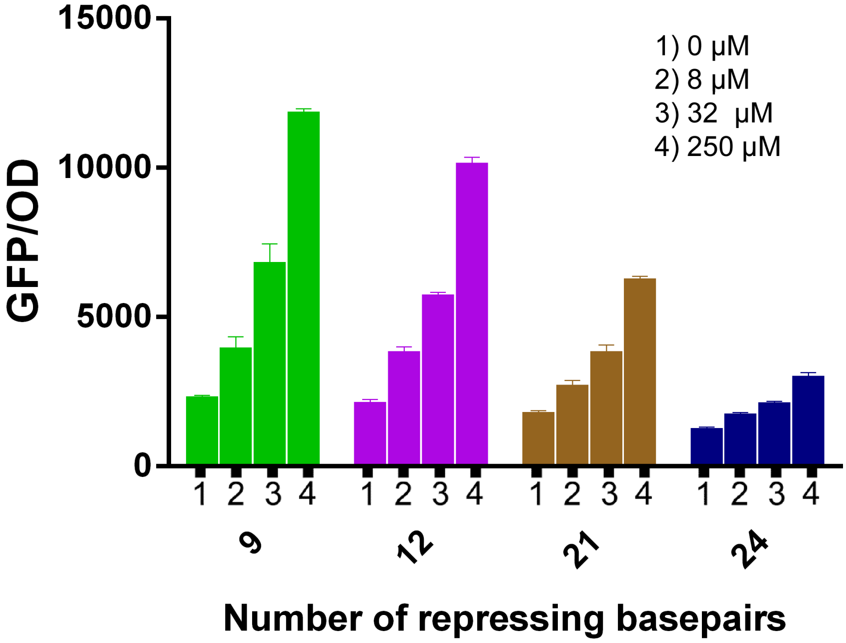

Figure S2: **Additional ribo-attenuator screening data:** Response of ribo-attenuators not used in the main paper to varying levels of induction by 2-aminopurine. This experiment was performed using the above ribo-attenuators (which were designed to have hairpins consisting of 9, 12, 21, and 24 repressing bp respectively), as well as Att1 (3 repressing bp), Att2 (4 bp), Att3 (6 bp), Att4 (15 bp), and Att5 (18 bp), for which data is presented in Fig. 4. Att1-5 were selected from the nine candidates to provide a wide range of induction response behaviours. Error bars indicate standard deviation of measurements for biological triplicates.

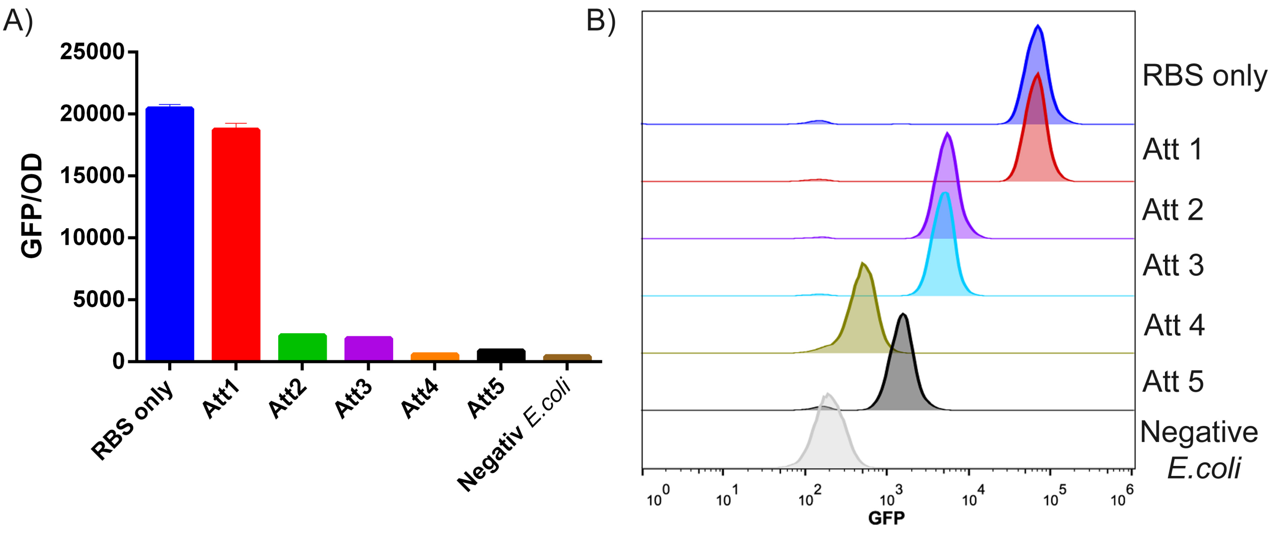

Figure S3: **Repressive effects of ribo-attenuators without riboswitch inclusion:** Each attenuator was introduced between the tetracycline promoter and sfGFP to assess its basal expression rate in the absence of an upstream riboswitch. A positive control (RBS only) was created by including only the Common RBS region (Fig. 2, Table S2) without the upstream repressing region. Introduction of the repressing regions resulted in a reduction in expression on a population (A) and single cell (B) level, though only a minor reduction was demonstrated by Att1 due to its weak hairpin. Error bars indicate standard deviation of measurements for biological triplicates.

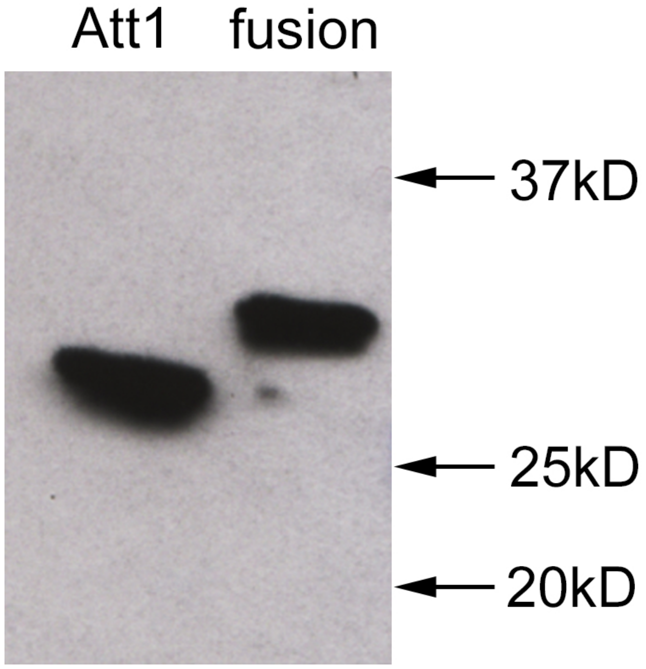

Figure S4: **sfGFP size analysis:** Immunoblot of sfGFP translated from the Adda riboswitch with the Att1 ribo-attenuator, and as a direct fusion to the first 150 bp of the riboswitch's working ORF (eGFP). The effectiveness of the transcriptionally coupled junction **TAA**TG is observed in the approximate 5 kD difference between constructs, demonstrating that the attenuator separates the introduced gene of interest from the 150 bp fusion. Immunobloting was performed as described in the materials and methods using a monoclonal GFP antibody (Clonetech).

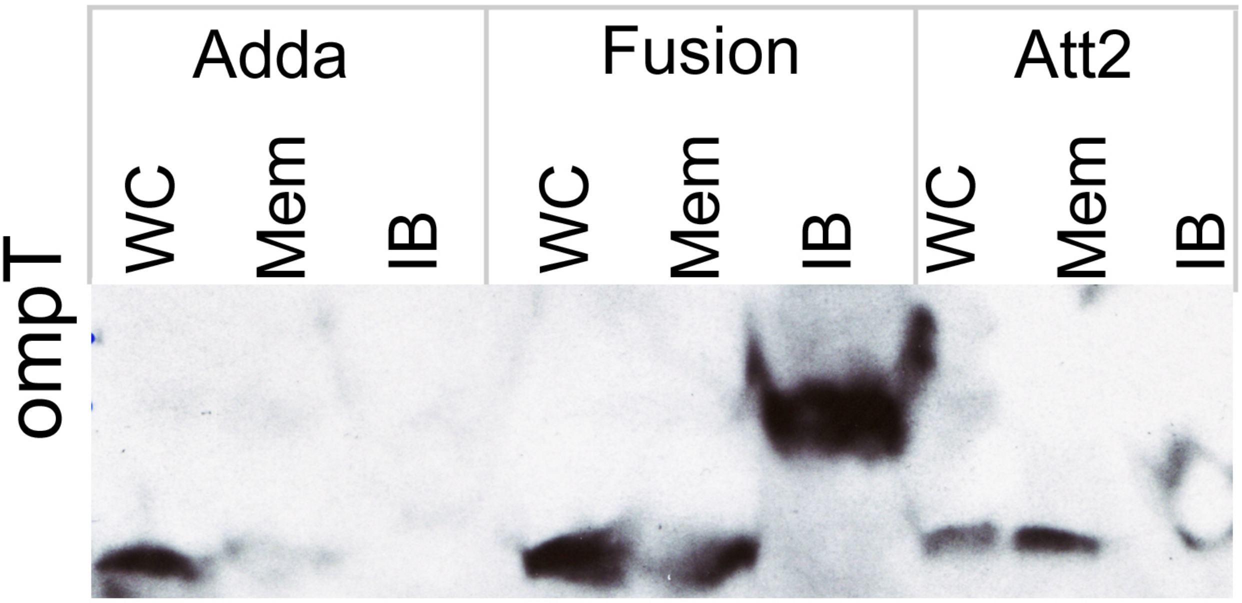

Figure S5: **OmpT targeting analysis:** Immunoblot of strep-tagged OmpT expressed from the Adda riboswitch directly, as a fusion with the first 150 bp of the Adda system's working ORF, and with Att2 isolating OmpT from the fusion domain. To assess targetting of OmpT, membranes (Mem) and inclusion bodies (IB) were separately dissolved on the blot demonstrating that the fusion approach to expression leads to a prevalence of insoluble inclusion bodies, whereas the ribo-attenuator system promotes proper membrane targetting. Whole cell (WC) fractions were included to demonstrate the basal expression of the system.

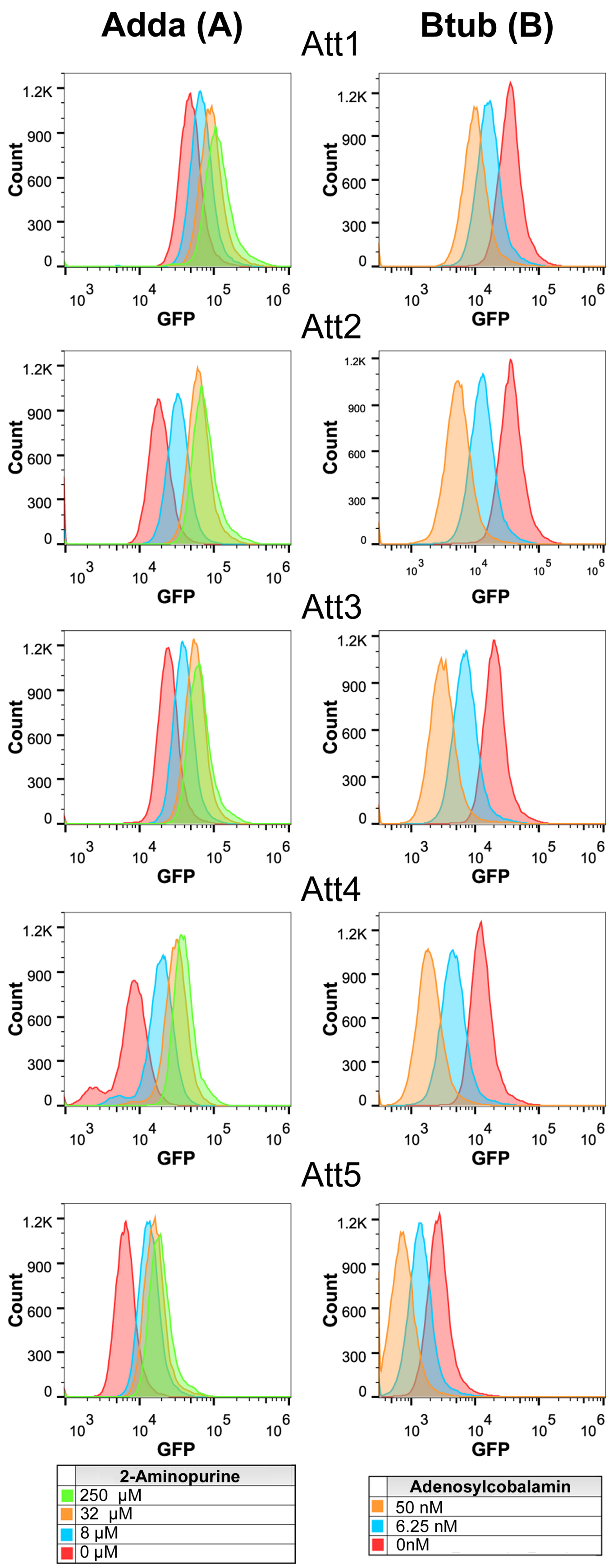

Figure S6: **Full single cell analysis for all ribo-attenuators:** Single cell analysis for both Adda and Btub riboswitches over reported inducer concentrations for all ribo-attenuators used.

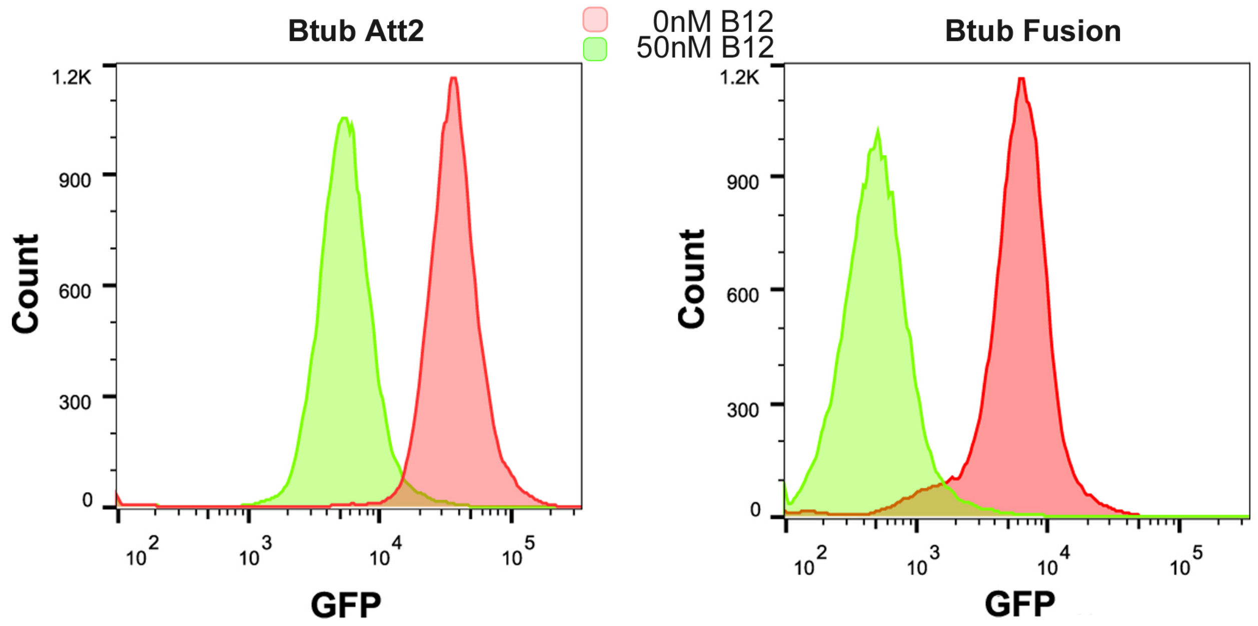

Figure S7: **Single cell comparison for the induced/uninduced Btub systems** Overlay showing induction response for Att2 and the fusion construct in the absence of inducer (red) and the presence of 50nM B12 (green).

Strain or Plasmid	Description	Reference or Source
E. coli		
DH5 αZ1	Expression and cloning host	[1]
Plasmids		
pSB50C1	Adda Riboswitch:150 bp ORF:sfGFP fusion	This study
pSB50C2	Adda Riboswitch:sfGFP	This study
pSB50C3	Btub riboswitch:150 bp ORF:eGFP fusion	This study
pSB50C4	Btub riboswitch eGFP	This study
pSB50C5	Adda riboswitch Att1:sfGFP	This study
pSB50C6	Adda riboswitch Att2:sfGFP	This study
pSB50C7	Adda riboswitch Att3:sfGFP	This study
pSB50C8	Adda riboswitch Att4:sfGFP	This study
pSB50C9	Adda riboswitch Att5:sfGFP	This study
pSB50C10	Btub riboswitch Att1:eGFP	This study
pSB50C11	Btub riboswitch Att2:eGFP	This study
pSB50C12	Btub riboswitch Att3:eGFP	This study
pSB50C13	Btub riboswitch Att4:eGFP	This study
pSB50C14	Btub riboswitch Att5:eGFP	This study
pSB50C15	(RBS only) Att0:sfGFP	This study
pSB50C16	Att1:sfGFP	This study
pSB50C17	Att2:sfGFP	This study
pSB50C18	Att3:sfGFP	This study
pSB50C19	Att4:sfGFP	This study
pSB50C20	Att5:sfGFP	This study
pSB50C21	Btub riboswitch:ColE9, immunity	This study
pSB50C22	Btub riboswitch:150 bp fusion:ColE9, immunity	This study
pSB50C23	Btub riboswitch:150 bp Att2:ColE9, immunity	This study
pSB50C24	Adda riboswitch:OmpT	This study
pSB50C25	Adda riboswitch:150 bp fusion:OmpT	This study
pSB50C26	Adda riboswitch:150 bp Att2:OmpT	This study

Table S1: List of strains and plasmids used in this work.

Attenuator	Repressing region	RBS region	ΔG kcal/ mole	N. repressing base pairs
Att1	CCT	AAGGGCCCAAGTTCACTTAAAAAGGAGATCAACTA	-2.3	3
Att2	-	AAGGGCCCAAGTTCACTTAAAAAGGAGGTCCTCTA	-7.4	2 in and 4 after RBS core
Att3	TCTCCT	AAGGGCCCAAGTTCACTTAAAAAGGAGATCAACTA	-8.2	6
Att4	AATGATCTCCTTTTTAAA	AAGGGCCCAAGTTCACTTAAAAAGGAGATCAACTA	-20.6	15
Att5	AATGATCTCCTTTTTAAGTGT	AAGGGCCCAAGTTCACTTAAAAAGGAGATCAACTA	-25.1	18

Table S2: List of ribo-attenuator sequences and predicted structural free-energy. For lowest free energy structures see Supplementary Fig. S1

References

[1] R. Lutz and H. Bujard, "Independent and tight regulation of transcriptional units in escherichia coli via the lacr/o, the tetr/o and arac/i1-i2 regulatory elements." *Nucleic acids research*, vol. 25, pp. 1203–10, 1997.

[2] J. S. Reuter and M. D. H, "RNAstructure: software for RNA secondary structure prediction and analysis," *BMC Bioinformatics*, vol. 11, no. 129, 2010. [Online]. Available: http://rna.urmc.rochester.edu/RNAstructureWeb/Servers/Fold/Fold.html
